# Characterization of Amorphous Ibrutinib Thermal Stability

**DOI:** 10.1021/acs.oprd.4c00299

**Published:** 2025-01-07

**Authors:** Dan Trunov, Jan Ižovský, Josef Beranek, Ondřej Dammer, Miroslav Šoóš

**Affiliations:** † Department of Chemical Engineering, University of Chemistry and Technology, Technická 3, Prague 6, Dejvice 166 28, Czech Republic; ‡ Zentiva, k.s., U Kabelovny 130, Prague 10 102 00, Czech Republic

**Keywords:** drug degradation kinetics, drug polymerization, comparing methods of amorphization, mass spectrometry, Zimm plot

## Abstract

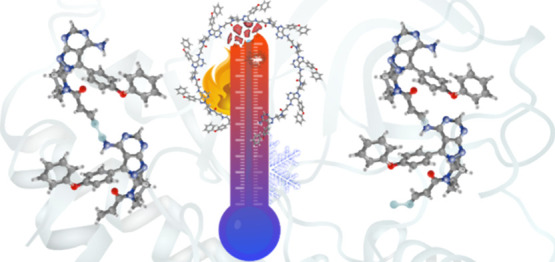

The choice of method for drug amorphization depends on various factors, including the physicochemical properties of the active pharmaceutical ingredients, the desired formulation, and scalability requirements. It is often important to consider a combination of methods or the use of excipients to further enhance the stability and performance of the amorphous drug. This study presents a comparison of techniques including melt quench, hot melt extrusion, solvent evaporation, ball milling, and lyophilization used for the preparation of amorphous ibrutinib. The amorphous material was thoroughly investigated using numerous techniques to examine changes in the physicochemical properties, stability, and degradation pathways of the drug product. During the examination, the temperature was discovered to be a key parameter for controlling the solubility and permeability of ibrutinib, which is influenced by the presence of the degradation product. We found that this degradation product could potentially polymerize and increase the molecular weight. The quantity, polymerization rate, and structure of the impurity can be regulated by the temperature variation during the amorphization processes. Additionally, the molecular weight of the degradation product was determined using Zimm plot analysis, which appeared for the first time in the literature for molecules of this category.

## 1 Introduction

Ibrutinib (IBR) is a potent covalent irreversible inhibitor of Bruton’s tyrosine kinase (BTK) and demonstrates significant activity against several B-cell malignancies, including mantle cell lymphoma, chronic lymphocytic leukemia, and chronic graft-versus-host disease.^1−4^ The drug covalently binds to the CYS-481 residue at the active site of BTK, which is stable for 24 h after administration.^5^ In addition to BTK, IBR also shows various binding affinities to other kinase inhibitions, such as the epidermal growth factor receptor family or the tyrosine kinase enzyme (JAK3).^6^ Currently, there is undergoing evaluation of IBR efficacy against other tumors.^4,7−10^ The drug is currently administered orally with a very low bioavailability of approximately 3%, thus resulting in extremely low absorption efficiency.^11^ Such low bioavailability is due to the combination of low solubility and first-pass metabolism of IBR. As a result, IBR is produced in capsules with a dose of 140 mg per capsule, with the highest dosage for treatment of 560 mg per day. IBR has a pH-dependent solubility profile where it easily dissolves in the acidic pH of the stomach. In contrast, it has low solubility at elevated pH, which might result in drug precipitation in the intestine,^12,13^ which causes gastrointestinal side effects and increases drug toxicity.^14^

Traditional crystalline forms of drugs often face challenges in achieving optimal solubility and bioavailability.^15^ To overcome this limitation, various pharmaceutical methodologies have been used to modify the solubility and dissolution properties of poorly water-soluble drugs using a unimodal component. The most common and adaptable strategies include cosolvents precipitation,^11,16^ salt formation,^17^ particle size reduction,^18^ amorphization technique,^19^ or polymorphic screening.^20^ Among them, the preparation of amorphous drug forms involves transforming crystalline drug structures directly into amorphous states, resulting in higher-energy states and enhanced molecular mobility. Depending on the physicochemical properties of the drug and formed amorphous form, these can be stable for the desired time or require the usage of excipients which would prevent recrystallization.^21^ The preparation of such active pharmaceutical ingredient (API) solid forms requires conversion of the crystalline drug into its amorphous form relying on the thermal, chemical, or mechanical energy input. There are several methods used for drug amorphization, each with its own advantages and limitations. Spray drying,^22^ hot melt extrusion,^23^ ball milling,^24^ melt-quench,^25^ solvent evaporation,^26^ lyophilization,^27^ and antisolvent precipitation^26^ techniques refer to most functional methods in the field of pharmaceutics. Each of these procedures presents unique challenges and requires specific optimization processes.^28^

**Figure 1:**
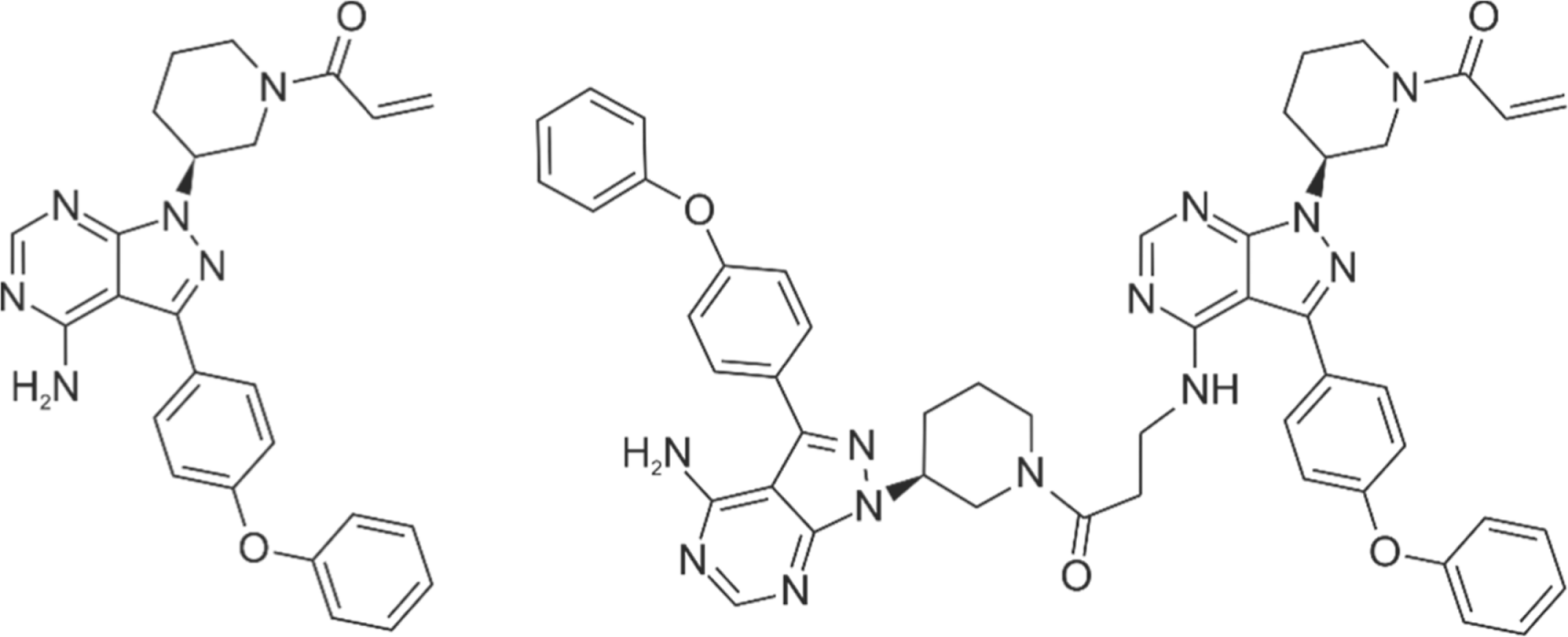
Chemical structures of IBR and its thermal degradation product impurity di-piperidine.

Nevertheless, among the promises of these methods, a critical concern about drug stability may arise due to the implementation of thermal, chemical, or mechanical energy. These products encompass a spectrum of unwanted chemicals that, even in small amounts, can affect the safety of chemical substances.^29^ Thermal degradation is a primary concern as most unpredictable results range from minor impurities to potentially genotoxic substances.^30^ Identification, quantification, and understanding of the source of these impurities require the application of rigorous analytical techniques and a deep understanding of the underlying chemistry. In the case of IBR, there are already several identified impurities^31,32^ as well as their degradation pathways.^33^ However, these publications do not fully reveal the true extent of thermal degradation products of IBR (see Figure 1) into ibrutinib di-piperidine (see Figure 1) and potential formation of other oligomers. In fact, the polymerization phenomenon is widely known in the pharmaceutical industry during the manufacturing process and storage of APIs. Such a kind of degradation mechanism has been reported for drugs like azetidine^34^ or penicillin.^35^

In our study, we employed commonly used methods, i.e., including melt quench, hot melt extrusion, solvent evaporation, ball milling, and lyophilization, to prepare amorphous solid dispersions of IBR (without any additives, i.e., polymers or plasticizers), covering a wide temperature range from room temperature to 240°. Such a broad temperature range allowed us to identify a suitable amorphization technique for the IBR molecule, resulting in enhanced solubility–permeability characteristics while minimizing the risk of impurities formation. Characterization of the physicochemical properties for all freshly prepared amorphous forms of IBR was done by X-ray powder diffraction (XRPD), nuclear magnetic resonance spectrometry (NMR), thermal gravimetric analysis (TGA), and Fourier-transform infrared (FT-IR) spectroscopy. Furthermore, solubility, permeability, and degradation patterns of IBR under thermal treatment were thoroughly investigated using UV–vis spectroscopy and liquid chromatography in combination with mass spectrometry (UPLC–MS). The dynamic light scattering (DLS) technique was employed to examine the maximum molecular weight of the degradation product.

## 2 Experimental Part

### 2.1 Materials and Reagents

IBR form A as an API was kindly provided by Zentiva, k.s. (Prague, Czech Republic). Ammonium dihydrogen phosphate, sodium chloride, and sodium acetate used in the dissolution medium were purchased from PENTA (Czech Republic). Solvents, such as dimethyl sulfoxide (DMSO), acetonitrile-*d*_3_, toluene, and ethanol, were obtained from Sigma-Aldrich. Analytical grades of ammonium acetate and acetic acid as the mobile phase for the chromatography were purchased from Sigma-Aldrich. LC–MS-grade acetonitrile was purchased from Honeywell (Czech Republic). Ultrapure-grade deionized water (specific conductivity 0.15 μS/cm, Aqual, Czech Republic) was used for the preparation of dissolution and dilution mediums.

### 2.2 Preparation of Solid Dispersions by Selected Methods

#### 2.2.1 Melt Quench

The glassy state of pure IBR was obtained after heating a total of 50 mg of crystalline form of IBR form A in a 50 mL ceramic crucible to 165 °C, 180 °C, 210 °C, or 240 °C for 15 min. Please note that the selected temperatures were in the range between the melting point of IBR (155 °C) and its decomposition temperature (280 °C). To avoid cooling from external sources, we wrapped the whole setup in aluminum. Subsequently, the melt was cooled by placing a crucible inside an ice-cooled water bath. The prepared glass samples were crushed with a mortar and stored in a refrigerator for further analysis. A summary of all used methods and their abbreviations is shown in Table 1.

**Table 1:** Summary of Used Methods and Their Abbreviations

full name methods	abbreviations	used temperature (°C)	solvent
melt quench	MQ 240	240	
melt quench	MQ 210	210	
melt quench	MQ 180	180	
melt quench	MQ 165	165	
hot melt extrusion	HME	165	
ball milling	BM	≈75	
solvent evaporation	ROT	50	methanol
lyophilization	FD	25	benzene
no methods	IBR	25	

#### 2.2.2 Hot Melt Extrusion

This method was selected as a scale-up technique from the melt-quench technique. A hot melt extruder Mini Extruder Hybrid 5-9 (Three-Tec GmbH, CH) with a feeding apparatus was employed to prepare amorphous IBR. The feed rate of the mixture was set at 3% per minute, while corotating twin screws were operated at constant screw rate 45 rpm and the maximum extrusion temperature was set to 165 °C. The extrudates were cooled to room temperature and crushed with a mortar. The prepared samples were stored in the refrigerator for further analysis. A summary of all used methods and their abbreviations is shown in Table 1.

#### 2.2.3 Solvent Evaporation

Solvent evaporation was performed in a rotary vacuum evaporator system (BÜCHI, CH) using methanol as a solvent. Amorphous IBR was obtained by dissolving 200 mg of crystalline IBR form A in 50 mL of methanol in a round-bottomed flask and quickly evaporating at a temperature of 50 °C using a pressure of 20.6 kPa. The prepared material was dried in a vacuum oven at 35 °C overnight. The prepared samples were stored in the refrigerator for further analysis. A summary of all used methods and their abbreviations is shown in Table 1.

#### 2.2.4 Ball Milling

The amorphous form of IBR was prepared via mechanical deformation in an oscillatory ball mill Retsch MM400 (Retsch, GE). Approximately 1 g of crystalline IBR form A was weighed into 25 mL ball milling jars. The powder was milled at 25 Hz for 5 min with one 20 mm diameter stainless steel milling ball. The prepared samples were stored in the refrigerator for further analysis. A summary of all used methods and their abbreviations is shown in Table 1.

#### 2.2.5 Lyophilization

A laboratory-scale Freeze-Dryer L10-55 PRO (Gregor, CR) was used for the lyophilization procedure. A 200 mg portion of IBR form A was dissolved in 50 mL of benzene inside of a round-bottom flask. The solution was then frozen using a deep freeze device at −40 °C for 6 h. A chamber pressure of 2 hPa was set for evaporation, and the temperature of the cold trap was set at −95 °C. The sublimation of the frozen solvent was carried out at room temperature for the following 24 h. This process resulted in a low-density snowlike solid substance that was stored in the refrigerator for further analysis. A summary of all used methods and their abbreviations is shown in Table 1.

### 2.3 Determination of IBR Concentration and Permeability by UV–Vis Spectroscopy

Agilent Cary 60 UV–vis (Agilent, USA) was used to measure the absorbance of IBR. Measurement was carried out with an optical stainless-steel probe with a path length of 20 mm. UV light from 200 to 500 nm was used with a scan speed of 400 nm/min for the analysis. The maximum absorbance of IBR was found at a wavelength of 260 nm, and then its concentration was calculated from the corresponding freshly prepared calibration curve.

Concentration measurements were performed in a pH 6.8 dissolution medium containing phosphate buffer saline (PBS) prepared according to the procedure in the literature.^36^ The solution was heated to 37 °C and stirred at 150 rpm using a magnetic stirrer bar with a diameter of 15 mm. These conditions were kept constant throughout the experiment. The quantity of IBR added to 300 mL of dissolution medium corresponds to 0.14 g/L. Please note that this amount is equivalent to the concentration of IBR in the Imbruvica single tablet formulation^37^ administered orally. The samples were collected at predefined time points (5, 30, 60, and 120 min), filtered with a 0.2 μm syringe filter, and diluted with ultrapure-grade deionized water, before analysis in UV–vis. All measurements were made in triplicates.

The same dissolution medium was used to determine the diffusion rate of the drug employing a cellulose membrane with a molecular weight cut off (MWCO) value of 12–14 kDa (approximately 2–2.1 nm). The donor and acceptor compartments were separated by a dialysis membrane with a diameter of 3 cm (area 7.069 cm^2^). The volume of one compartment is equal to 77 cm^3^. Each chamber was heated to 37 °C and stirred at 200 rpm using a magnetic stirrer bar with a diameter of 15 mm. Inside the donor compartment, the concentration of IBR was equal to 0.14 g/L. In contrast, the concentration of molecularly dissolved IBR in the acceptor compartment was determined by a UV–vis probe at predefined time points (every 1 min over the period of 60 min and every 2 min over the period of 120 min). The permeability of the drug was calculated from the transport of a small amount of drug across the membrane, where the flux *J* can be described as
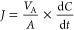
1Here, the flux depends on the changes in drug concentration over a specific period of time  in the acceptor compartment with volume *V*_A_ across the MWCO membrane with a specific area *A*.^38,39^

### 2.4 Ultra Performance Liquid Chromatography–Mass Spectra Studies (UPLC–MS)

All samples were analyzed with the Agilent 1290 Infinity II LC system (Agilent, USA) with a quaternary pump, autosampler, column oven, diode array detection (DAD), and single quadrupole MS detector operated with an electrospray ionization (ESI) source. The separation and identification of compounds were carried out on an ACQUITY UPLC BEH C18 column (1.7 μm, 2.1 mm × 100 mm) using a gradient mode of eluent A (ammonium acetate 20 mM, pH 6.0) and eluent B (acetonitrile) with a flow rate of 0.3 mL/min. Eluent A was freshly prepared by sonication and filtration by using a 0.2 μm PTFE syringe filter. The gradient mode of elution was set for [time in minutes (*t*_min_)/% of eluent B]: 0/20, 10/70, 14.9/70, and 15/20. The total run time of one measurement was 15 min. The column and autosampler temperatures were set at 45 and 10 °C, respectively. The injection volume was 5 or 10 μL, and the analyte detection was carried out at a wavelength of 215 nm. The MS analysis of IBR and its degradation product was carried out by using the MS detector. The analysis was carried out in positive ion mode using an ESI source at an operating voltage of 3 kV. The capillary temperature was set at 350 °C with a gas flow rate of 600 L/h, and detection was carried out in the range of mass spectra of 50 up to 1000 *m*/*z*. The IBR mass equal to 441 *m*/*z* was used for its detection. The retention time of IBR was found to be 9.74 min. The optimized chromatographic condition was obtained from the publication of Mehta et al.^33^ All parameters used are summarized in Table 2.

**Table 2:** Summary of the Used Parameters for UPLC–MS

column	ACQUITY UPLC C18, 100 × 2.1 mm, 1.7 μm
flow rate	0.3 mL/min
DAD detection	215 nm
column temperature	45 °C
autosampler temperature	10 °C
volume of injection	5 μL
ion	positive
voltage	3 kV
capillary temperature	350 °C
mass spectra	50–1000 *m*/*z*
mass detection	441 *m*/*z*
gas flow	600 L/h
eluent A	20 mM ammonium acetate, pH 6.0 adjusting CH_3_COOH
eluent B	acetonitrile
	0 min 20% B
	10 min 70% B
gradient elution time	14.9 min 70% B
	15 min 20% B

The powder sample containing IBR was dissolved with the acetonitrile:water (50:50 v/v %) diluent for 1 h to obtain the nominal concentration of 100 μg/mL. A PTFE syringe filter of 0.2 μm was used to filter the solutions prior to the analysis in UPLC–MS. A blank solution of both mobile phases was prepared using the same procedure. The IBR concentration was calculated from the corresponding calibration curve with relevant linearity (*R*^2^ > 0.999). All measurements were made in triplicates.

### 2.5 X-ray Powder Diffraction (XRPD)

The XRPD patterns of the prepared solid dispersions were obtained using the PANalytical X’Pert^3^ powder X-ray diffractometer (PANalytical, Holland). The diffractometer was operated in diffraction mode 2Θ with the copper radiation source Cu Kα (λ = 1.544 Å), operated at a voltage of 40 kV/30 mA. The powder samples were compressed as a thin layer inside a 10 mm sample holder. Each diffractogram was recorded in the 2Θ range of 5 to 40° with a step size of 0.001°.

### 2.6 Nuclear Magnetic Resonance Spectrometry (NMR)

Approximately 50 mg of solid sample was dissolved in 10 mL of deuterated acetonitrile-*d*_3_ in a 5 mm NMR tube and measured at room temperature. The NMR spectra were obtained using an Avance III (Bruker, GE) operating at 500 MHz equipped with an inverse detection 5 mm ^1^H double resonance broadband probe with a *z* gradient. The ^1^H NMR spectrum was acquired by using TopSpin 4.1.1 software.

### 2.7 Thermal Gravimetric Analysis (TGA)

The TGA data was collected using a Pyris TGA 1 (PerkinElmer, USA). Approximately 5 to 8 mg of sample were weighed in a covered aluminum pan and measured in air gas flow. Measurements were made at a temperature heating rate of 10 °C/min in the range of 25 to 500 °C.

### 2.8 FT-IR Spectroscopy

FT-IR spectroscopy was performed on a Nicolet Nexus 670 (Thermo Fisher Scientific, USA) equipped with a deuterated triglycine sulfate (DTGS) detector and a ZnSe attenuated total reflection accessory. A total of 128 scans (using transmission mode) were performed in the spectral region 4000–650 cm^–1^ using a resolution of 4 cm^–1^ at ambient temperature. Before measurements, the prepared samples were ground and dried at 35 °C overnight.

### 2.9 Molecular Weight Analysis Using DLS

To determine the characteristic molecular weight of the prepared samples, we conducted standard dynamic/static light scattering measurements using a 3D LS spectrometer from LS Instruments (Fribourg, CH) using 2D pseudocross correlation. The intensity of the scattering molecules was measured at various scattering angles in the range of 50 to 130° with a step size of 10° and various concentrations of IBR. The laser and scattered intensities were automatically adjusted by the equipment. The wavelength of the Cobolt modulated DPSS laser was 660 nm. All measurements were carried out at 25 °C.^40^ The amorphous IBR prepared by MQ was dissolved in doubly filtered DMSO solvent using a 0.45 μm PTFE syringe filter at a maximum concentration of 1 wt %. The prepared solution was sonicated for 30 min using a sonication bath. Then, solutions with different concentrations were obtained by the dilution of more concentrated solutions.

The data analysis for the extraction of the average molecular weight () was done in the software LSI Zimm Plot (version 1.1.1.40). The Zimm plot represents an easy graphical way to perform data fitting using an excess Rayleigh ratio by a solvent. The representation of data as a plot on the scattering angle corresponds to the equation
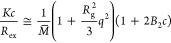
2where the scattering vector *q*(41) is defined as follows
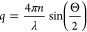
3

with *n* being the refractive index, and Θ represents the scattering angle. In eq 3, *K* is the electromagnetic constant, *c* is the concentration of the IBR solution, and *R*_ex_ is the excess Rayleigh ratio contribution of the material chains to the scattered light intensity. The electromagnetic constant *K* includes information about the specific refractive index , which was measured separately using a dipping refractometer CARL ZEISS with a precision of 1 × 10^–5^. The specific refractive index was extracted from the refractive index measurements versus the concentration of dissolved IBR (Supporting Information Figure S1).

In the situation where the extrapolation is performed at *q* = 0, it is possible to obtain a second virial coefficient *B*_2_, corresponding to the repulsive particle interactions.^42,43^ Furthermore, by extrapolating the data to *c* = 0, the slope of *Kc*/*R*_ex_ vs *q*^2^ + *B*_2_*c* corresponds to the radius of gyration *R*_g_. The molecular mass of the characterized molecule could be extracted by performing linear interpolation of the measured data for *q* ≠ 0 for all concentrations of IBR.

## 3 Results and Discussion

### 3.1 Physicochemical Properties of the Crystalline and Amorphous Forms of IBR

**Figure 2:**
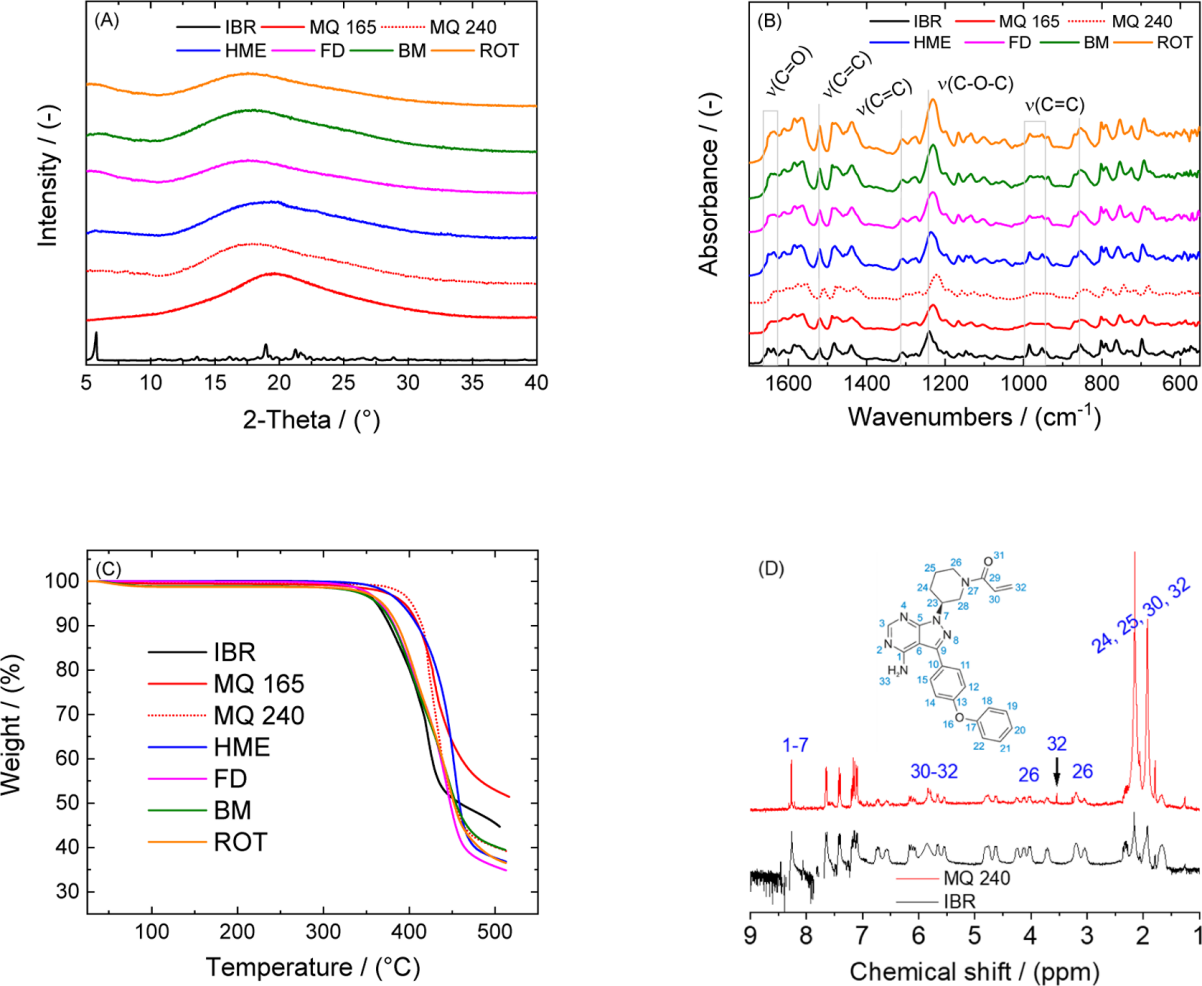
Solid state properties of the drug as well as crystalline and solid dispersions forms of IBR prepared by selected methods of amorphization: (A) XRPD diffractograms; (B) FT-IR spectra (the gray lines represent the characteristic absorption bands of the IBR compound); (C) TGA thermograms; (D) ^1^H NMR spectrum of the crystalline form of IBR and sample prepared by method melt quench.

Comparison of the diffractograms measured for samples prepared by various procedures discussed above is summarized in Figure 2A, together with the pattern measured for crystalline IBR form A. As can be seen, the IBR form A pattern has strong characteristic diffraction peaks at 5.78, 13.56, 16.12, 18.96, 21.26, 21.74, 22.43, 24.6, and 28.83° (2θ).^44^ In contrast, all selected amorphization methods, i.e., melt quench, hot melt extrusion, solvent evaporation, ball milling, and lyophilization, exhibited only a broad signal without the presence of any characteristic peaks typical for fully amorphous solid forms. Additionally, the melting endothermic peak of the crystalline form of IBR was found to be 159 °C, while its amorphous state, prepared by all amorphization techniques, was equal to 59–61 °C. The full thermograms from modulated dynamic scanning calorimetry are summarized in Supporting Information (Figure S2). This information fully confirmed the absence of the crystalline forms of the API.

Due to the stable character of the prepared amorphous forms of IBR, the FT-IR measurements were performed for identification of any amorphous changes or possible interaction between IBR functional groups. A summary of all FT-IR spectra is presented in Figure 2B. The spectrum of crystalline IBR form A shows the main functional characteristic groups. The strong characteristic peak of two connected aromatic cycles appeared at 1239 cm^–1^ for the ν(C–O–C) stretching frequency. Strong peaks are also observed at wavenumbers of 1651 and 1636 cm^–1^, which can be assigned to the ν(C=O) stretching frequency. The series of other peaks at wavenumbers 1521, 1308, 983, 955, and 855 cm^–1^ can be assigned to the ν(C=C) stretching frequency of aromatic cycles or the double bond at the end of the molecule (see Figure 1). A slightly different situation occurs when IBR is exposed to thermal treatment during the amorphization process. In particular, it was observed that the characteristic peaks at wavenumbers of 1651, 1636, 983, 955, and 855 cm^–1^ were shifted or eliminated. Changes in infrared shift or shape in specific bands may indicate a drug–drug interaction associated with ν(C=C) or ν(C=O) stretching frequency. The broader bands obtained at range wavenumbers 1000–950 cm^–1^ may also reflect the existence of weak intermolecular interactions, such as van der Waals, electrostatic, hydrophobic, or π interactions.^45^ This indicates that during heating processes, IBR undergoes a reaction that may result in the formation of a complex structure as well as possible appearance of the degradation products. Additionally, long-term thermodynamic stability was tested by FT-IR measurements for all prepared samples to assess the physical state of IBR. FT-IR spectroscopy revealed that all samples were amorphous over 12 months in normal storage conditions by observing a strong vibrational similarity at stretching frequencies 1651 and 1636 cm^–1^. This confirms the long-term stability of all amorphous solid forms of IBR.

Since all selected amorphization procedures may induce the thermal degradation of drug substances, TGA measurement was carried out to clarify the behavior of the drug under nonisothermal conditions. All TGA thermograms are summarized in Figure 2C. In all samples was observed a small weight loss at the beginning of the experiment, which corresponds to evaporation of adsorbed water. As can be seen, IBR form A has two different steps of thermal degradation. The first weight loss is found from 320 °C, while the second stage is observed from 420 °C onward. The latter signal corresponds to sample carbons burning out (Section 2.7). In the amorphous solid forms prepared by thermal treatment of the samples for which the thermal stress was greater than 100 °C, the first weight loss shifts to the higher value up to 375 °C, which is possible to observe for samples MQ 180, MQ 240, and HME. Furthermore, as suggested by the data, there is a systematic shift of the TGA signal to higher temperatures for higher-temperature treatment during melt quench. As suggested by Zhu et al.,^46^ changes in thermogravimetric shifts may indicate the presence of higher molecular weight impurities upon IBR thermal treatment. This is in line with the results of FT-IR analysis, where the drug–drug interaction associated with the ν(C=C) or ν(C=O) stretching frequency was observed. In contrast, amorphous samples prepared with methods where the temperature was below 100 °C did not reveal the shift of the TGA signal compared to the crystalline form of IBR form A.

Since TGA measurements revealed a possible change in the molecular structure during the thermal treatment, further information about the physical stability of sample MQ 240 (highest temperature used during melt quench) was collected by NMR analysis. Please note that to achieve intensity above the detectable limit, measurements were conducted in a higher volume of organic solvent, and dissolution was performed in a sonication bath for a period of 60 min. The ^1^H NMR spectrum showed 9 aliphatic protons and 10 aromatic protons, and the spectra are comparable to that shown by Vajjha et al.^31^ Summaries of the results are plotted in Figure 2D and are also reported in Table S3. Parallel to the basic protons observed for IBR, in the MQ 240 sample was observed a higher intensity of extra hydrogen atoms at chemical shift 3.54 ppm (C32 indicated by the arrow). Furthermore, a higher intensity was found at a characteristic atom position at 1.87, 2.22, and 2.45 ppm. Those positions correspond to the carbonyl atom C32 with a double bond at the end of the drug molecule. Nevertheless, for samples MQ 165, MQ 180, MQ 210, and HME, the intensity was below the detection limit of the equipment for quantitative analysis. Based on the previously mentioned results, we can conclude that exposing IBR to higher temperatures leads to the formation of thermal degradation impurities, e.g., ibrutinib di-piperidine (Impurity Di).^33^

### 3.2 Forced Degradation of IBR by Thermal Decomposition

**Figure 3:**
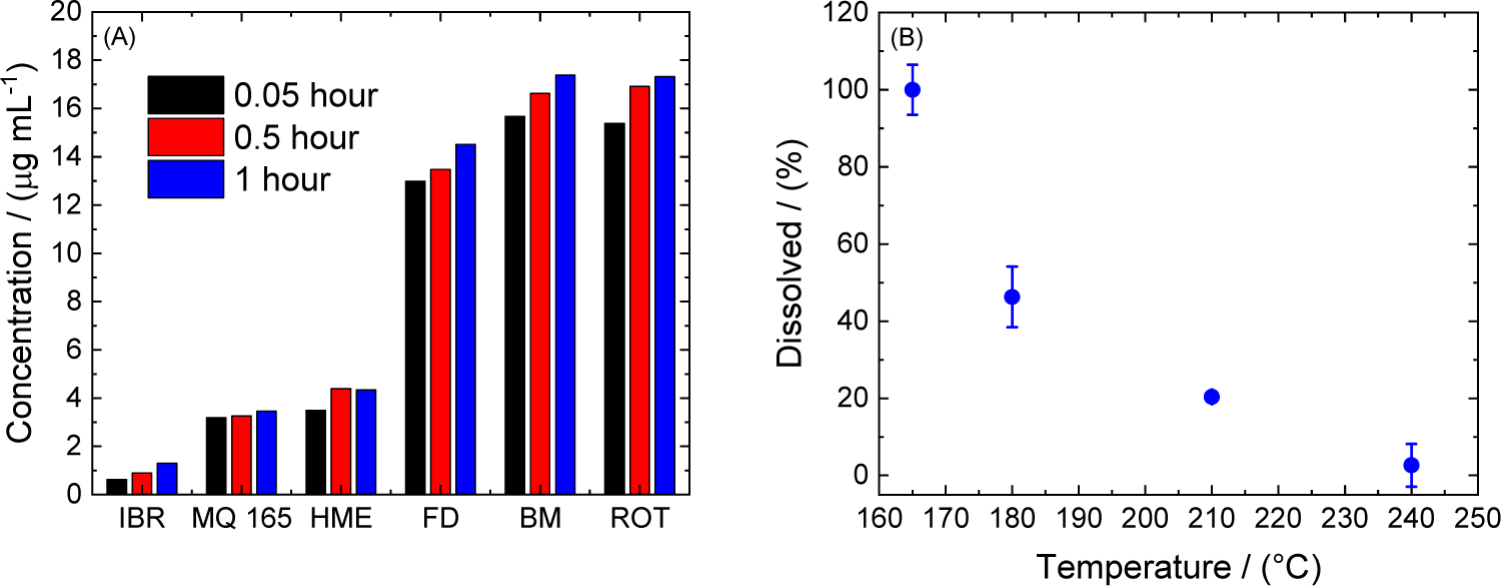
(A) Concentration kinetic of the dissolved amount of IBR versus the method of drug preparation. (B) Amount of the dissolved API in the normalized percentage for sample MQ 165 versus temperature of thermal treatment of the drug achieved using method MQ. The blue points correspond to time interval 60 min.

Since the dissolved amount of crystalline IBR form A exhibited a solubility of around 1.29 μg cm^–3^ in the PBS solution, in what follows, we compared the amount of dissolved drug for selected amorphized samples. As shown in Figure 3A, the improvement of IBR solubility is strongly dependent on the used amorphization method following the order IBR form A < MQ 165 < HME < FD < BM < ROT. In addition, there is a strong difference between methods utilizing high temperature above 100 °C compared to low-temperature methods. Samples MQ 165 and HME exhibit comparable solubility behavior, consistent with the overall trend observed in the data sets. Please note that for MQ 165 and HME samples was used the same maximum temperature during preparation. The solubility improvement by the methods BM and ROT was identical, based on the maximum dissolved amount achieved for the amorphous form due to the specific characterization procedure applied (see Section 2.3). Parallel to previously mentioned techniques, sample FD follows a similar trend with slightly lower concentration comparing samples BM and ROT. When considering conditions for amorphization, we can see that for all three methods, the temperature was significantly below minimum temperature for MQ or HME, i.e., 165 °C. Such results indicate that there is strong thermal instability of IBR when exposed to high temperatures. As shown in Figure 3B, applying a temperature of 180 °C leads to a decrease in solubility concentration by at least half compared to a temperature of 165 °C. On the other hand, when the temperature surpasses 210 °C, the concentration decreases by a factor of 5, followed by a reduction in the dissolved amount by up to 18 times for a temperature of 240 °C. This phenomenon can be explained by an addition reaction on the double bond at the end of the IBR molecule leading to the formation of high molecular oligomers.

**Figure 4:**
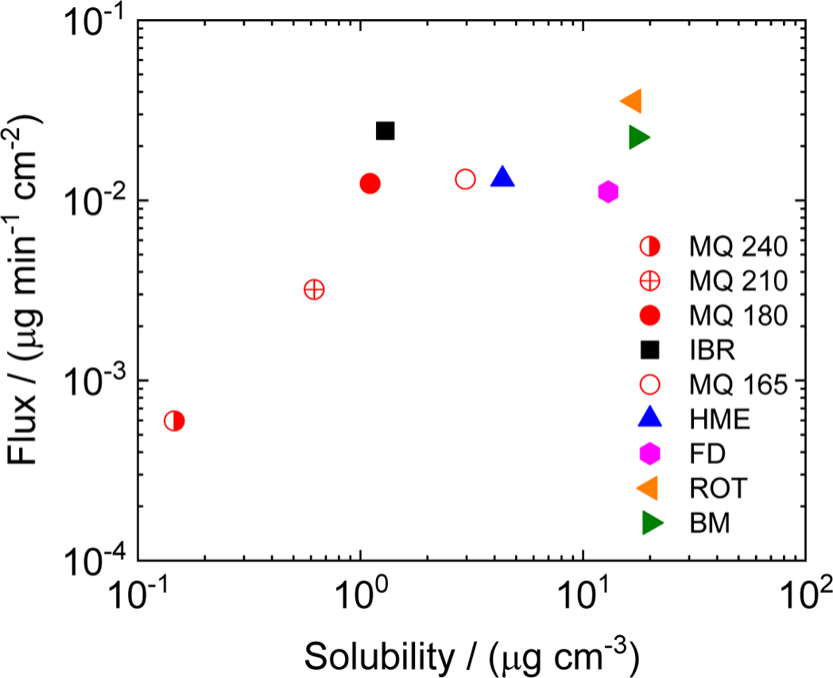
Illustration of the BCS diagram based on the obtained values between solubility and flux of the drug and its amorphous form.

As a result of the oligomerization reactions, the total chain length of the molecule is extended. Consequently, the diffusion coefficient is reduced. Therefore, we measured both the solubility and the flux of all prepared amorphous dispersion forms, and the results are summarized in Figure 4. As can be seen, samples MQ 210 and MQ 240 exhibit orders of magnitude lower permeability and solubility. These results confirm the presence of a larger quantity or longer chain length impurities of IBR, leading to a significant decrease in penetration rate. In parallel, both amorphous MQ 180 and crystalline IBR form A demonstrate similarities in their permeability–solubility interplay. The same assertion can be applied to samples MQ 165 and HME, where a similarity in permeability and solubility was also detected. Samples with maximum solubility corresponding to FD, BM, and ROT are also accompanied by higher flux values. In addition to the previously mentioned descriptions, the obtained flux values can be categorized into three regions. First, where the flux values are the lowest, below those of samples MQ 240 and MQ 210. The second region corresponds to samples MQ 180, MQ 165, HME, and FD, where the flux remains approximately constant. The final section pertains to IBR form A, BM, and ROT, where the highest flux values were obtained. Lower permeability values can be compensated by increasing solubility over a longer time interval, which is sufficient for IBR due to its inhibitory properties in the human body. As is evident from the introduced BCS-like diagram (Figure 4), drug impurities can be identified during the amorphization procedure, especially when the molecular weight of the final product increases. Therefore, depending on the selection of various membranes, the formation of various drug impurities during the preparation of amorphous solid dispersions, influenced by the solubility–permeability interplay, may be revealed.

**Figure 5:**
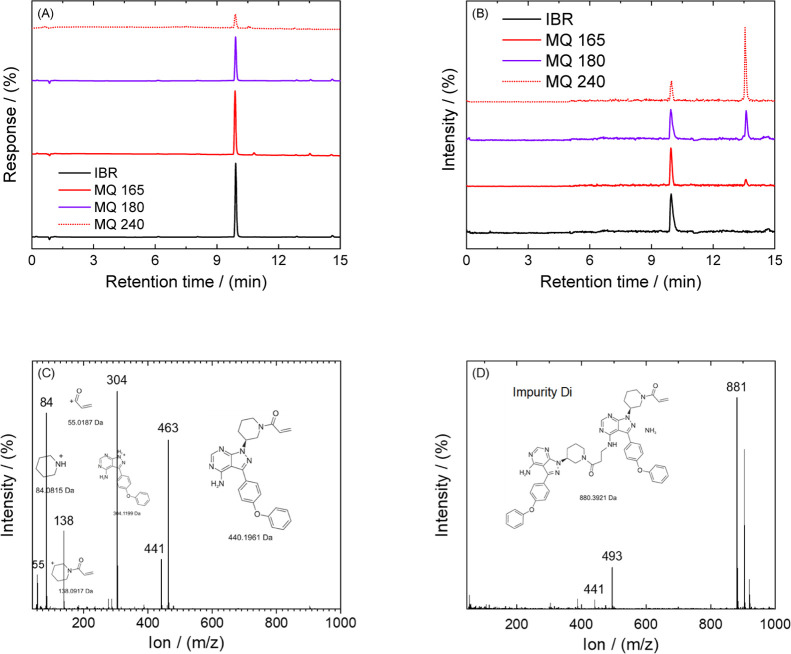
Example of measured chromatograms of the pure component IBR and its thermal degradation product separated by the detectors used: (A) DAD; (B) MS. Measured ions spectrograms and appropriate structure of molecules in the retention time interval: (C) 9.8 min; (D) 13.6 min.

Considering the complexity (higher values of the molecular weight) of the studied molecules, UPLC in combination with MS analysis was chosen for all of the prepared samples. When considering the analysis using the DAD detector (see Figure 5A), we are able to detect only molecules of IBR after chromatographic separation. As can be seen in Figure 5A, the peak was found at a retention time of 9.8 min and belongs to pure IBR. Therefore, samples MQ 165, MQ 180, and MQ 240 are identical to the previously mentioned sample when the peak was found at the equivalent retention time. In the case of the samples prepared by the melt-quench technique, for the same amount of injected IBR, the area under the curve was lower compared to IBR form A, indicating lower solubility in organic solvent, according to the preparation method (see Section 2.3). This means that besides IBR, a contribution of the IBR impurity is noticed. Meanwhile, by the MS detector (see Figure 5B), a second peak at a retention time of 13.2 min was detected. The IBR mass spectrum in positive ESI mode in acetonitrile solution with a retention time of 9.8 min is shown in Figure 5C. The principal peak at *m*/*z* around 441 corresponds to the protonated compound (IBR + H^+^). In total, five fragmentation patterns for IBR were observed and are shown in Figure 5C. The first fragment having *m*/*z* around 304 was obtained by the removal of N-substituted acryloyl from IBR. Furthermore, the patterns that have *m*/*z* around 84 and 55 correspond to the formation of piperidin-3-ylium and acrylaldehyde ions, respectively. A combination of two previously described patterns resulted in the formation of 1-acryloylpiperidin-3-ylium with *m/z* around 138. The final fragmentation pattern was obtained by removing acrylaldehyde from IBR and its protonated compound with the addition of eluent A having *m*/*z* around 463. For a retention time of 9.8 min, all fragmentation data are based on the IBR removal structure for the characteristics of individual ions and their combination. The fragmentation spectrum for Impurity Di with a retention time of 13.2 min is shown in Figure 5D. In addition to previously described patterns, where IBR and its fragmentation ions were found, the main characteristic pattern was discovered at the *m*/*z* position around 881, which corresponds to the dimer of IBR.

**Figure 6:**
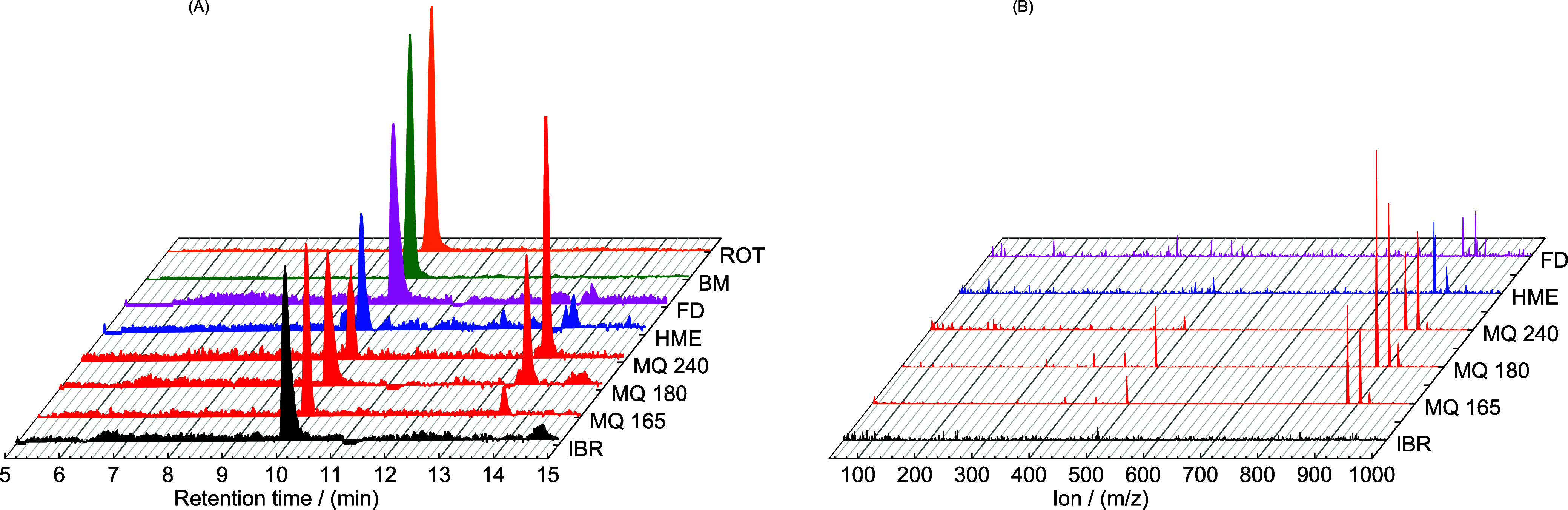
(A) Summarization of all MS chromatograms for different methods during preparation of amorphous solid dispersions. (B) Ions spectrograms of the selected methods at the retention time interval 13.6 min.

According to the discussion above, all samples prepared by the selected amorphization method were analyzed using the UPLC procedure in a combination with the MS detector, and corresponding MS chromatograms are presented in Figure 6A. As can be seen in all the prepared materials, the pure component of IBR can be observed at a retention time of 9.8 min. Parallel to the pure IBR compound, in samples from HME, MQ, and FD, it is possible to find also degradation product Impurity Di at a retention time of 13.6 min. These data are plotted in the form of *m*/*z* in Figure 6B, where also in this case, a compound with a *m*/*z* 881 that corresponds to the Impurity Di is also present. In parallel, pure IBR was also detected in the same position as discussed above (Figure 5C). While samples MQ and HME exhibit the formation of Impurity Di due to application of higher temperature, the presence of Impurity Di in sample FD indicates that this can be formed also by other mechanisms, i.e., chemical decomposition or handling in a cold environment according to the sample preparation procedure (see Section 2.2.5). Despite that, the amount of drug impurity in MQ 165, HME, and FD is significantly lower than in MQ 180 or MQ 240. Anyway, in all cases, the achievable preparation by thermal treatment exposure (low and high temperature) illustrates the presentation of the Impurity Di. In contrast, the samples prepared using ROT and BM are similar to pure IBR and do not show the presence of any Impurity Di.

### 3.3 Maximum Molecular Weight of IBR

**Figure 7:**
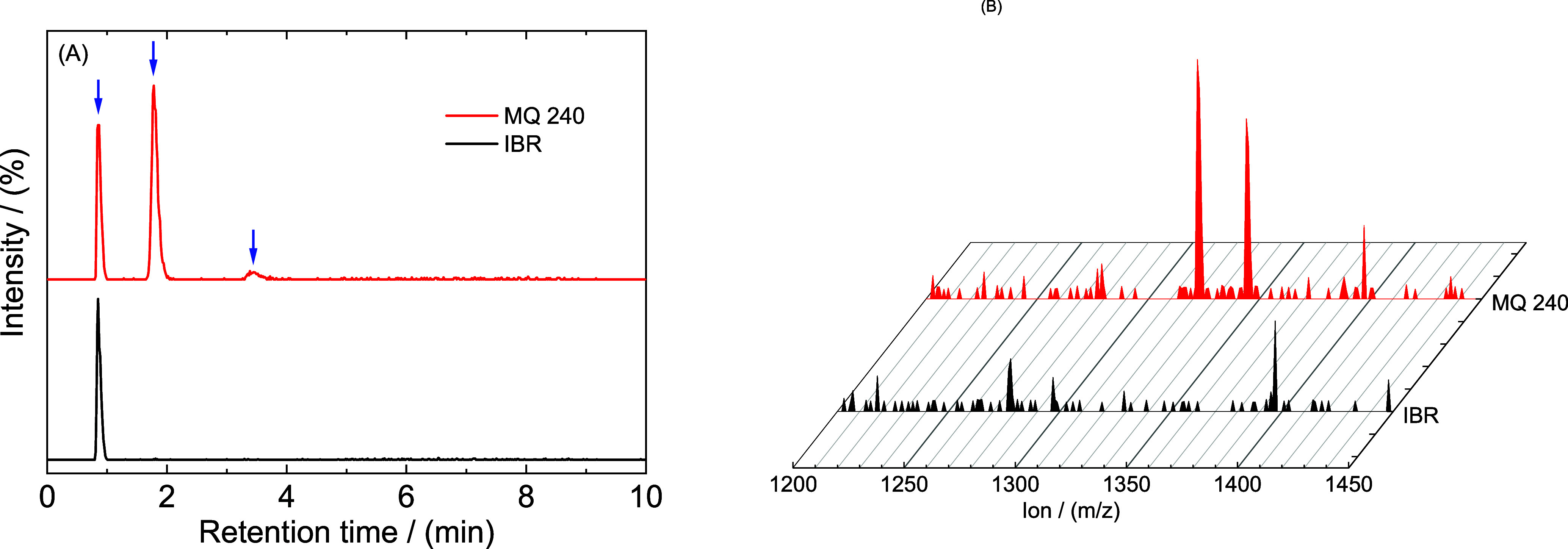
(A) MS chromatograms of IBR and samples MQ 240; the *x* axis is limited to the ions with molecular weights of 440, 881, 1322, and 1763 *m*/*z*. (B) Ions spectrograms of the selected method at the retention time interval 3.39 min.

Since the IBR molecules are able to polymerize, molecules can undergo further reactions to form larger oligomers.^47^ For separation of such oligomers, the modified procedure as described in Section 2.4 was used. For more accurate measurements, HPLC combined with quadrupole mass analyzers was applied, which shifted the retention time of IBR and oligomers to a shorter time. Considering the higher probability presenting of oligomers in exposure samples at high temperature, only sample MQ 240 was used for further analysis. As can be seen in Figure 7A, the total of three characteristic peaks was revealed based on the mass of the individual compound. Pure IBR and Impurity Di were identified at retention times of 0.85 and 1.78 min, respectively. A trimer was found at retention time 3.39 min and the mass spectrum is shown in Figure 7B. Parallel to the characteristic fragmentation pattern mentioned above of IBR and Impurity Di (see Figure 5C,D), the segmented pattern of the trimer was discovered at *m*/*z* 1322 by a protonated compound (three times IBR + H^+^).

**Figure 8:**
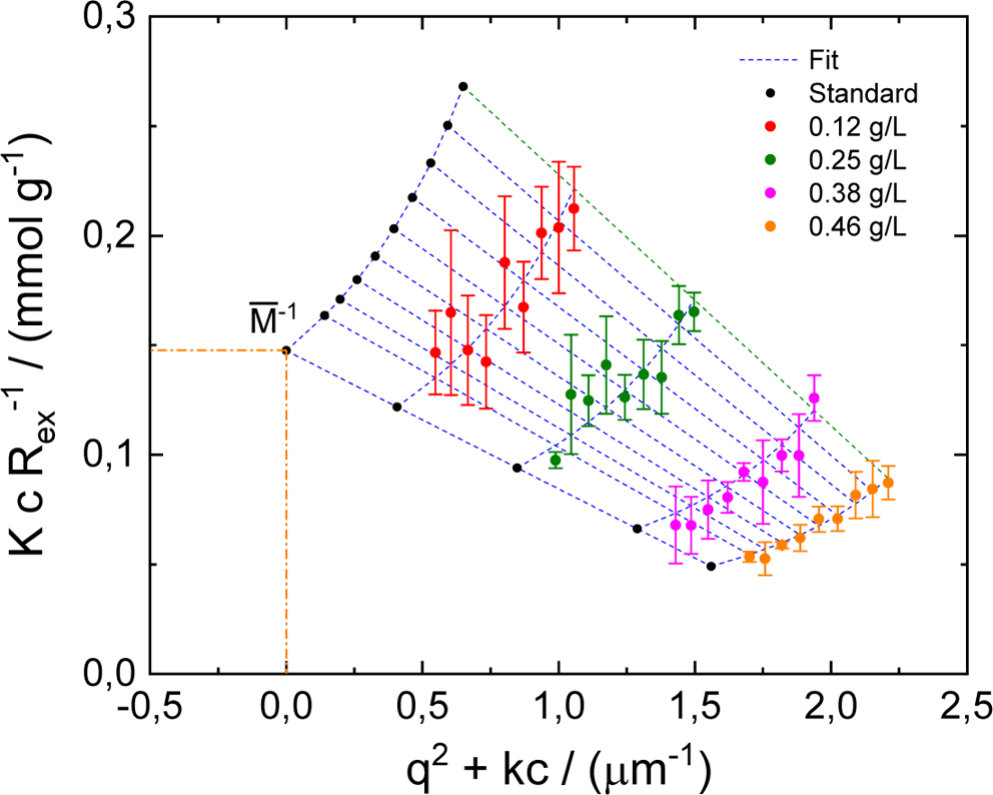
Zimm plot diagram of the dispersed sample MQ 240 in the DMSO solvent at various concentrations.

To demonstrate possible ranges of molecular weight for the presented oligomers, the light scattering measurement was performed using DLS equipment. Due to the higher probability of oligomers formation, sample MQ 240 was chosen for this analysis. The intensity data measured as a function of concentration and angle were demonstrated by the Zimm plot diagram and are shown in Figure 8. Since the specific refractive index for the sample MQ 240 dispersed in solvent DMSO was found to be around 0.89 ± 0.04 (Supporting Information Figure S1) as a consequence, the extrapolated molecular weight is in the range of 2072–2540 g/mol. Demonstrated values of molecular weights correspond to the IBR pentamer, which appeared during the polymerization steps. Presented results confirm that in the case of IBR and similar drugs with double bonds in the structure, there is high probability of oligomerization when exposed to higher temperature. Formation of high molecular weight oligomers will strongly affect drug solubility as well as permeability. Interestingly, when the drug was exposed to a chemical degradation procedure by using solvent benzene, sample FD underwent degradation pathways resulting in dimer formation, and the psychochemical properties are confirmed by Figure 2C.

## 4 Conclusions

In this work, a comparative study of several methods (i.e., melt quench, hot melt extrusion, solvent evaporation, ball milling, and lyophilization) to prepare an amorphous drug was carried out. It was found that when IBR is exposed to higher temperature, it undergoes degradation, resulting in formation of product impurities with a higher molecular weight (i.e., Impurity Di). This impurity, along with the amorphous form of IBR, influences the permeation and solubility properties of the drug in both organic and aqueous solvents. In particular, both the solubility and the permeability parameters decrease with an increasing process temperature. In parallel with Impurity Di, the polymerization reaction leads to an increase in molecular weight of impurities up to a pentamer due to the exposure higher temperature. This information has important implications for the stability, formulation, and behavior of IBR under specific conditions. The determination of the molecular weight of such oligomers, for the first time for this drug, was predicted using the Zimm plot diagram. As documented by our results, adjusting and maintaining the processing parameter temperature within specific ranges can have a significant impact on the formation of oligomers. This understanding can be instrumental in optimizing the manufacturing process to ensure the desired quality and properties of the final pharmaceutical product.

## Supporting Information Available

Details about the specific refractive index, modulated differential scanning calorimetry, NMR data collection, and the concentration of oligomers in the solution (PDF)
