# Supplementary material for: Characterization of Amorphous Ibrutinib Thermal Stability
Source: Org Process Res Dev. 2025 Jan 7;29(1):56–65. doi: 10.1021/acs.oprd.4c00299 (PMC11744788; doi:10.1021/acs.oprd.4c00299)
Supplement: Supplementary file 1 — op4c00299_si_001.pdf [file op4c00299_si_001.pdf]

Supporting information

Characterization of amorphous Ibrutinib thermal stability

Dan Trunov[a*], Jan Ižovský[a], Josef Beranek[b], Ondřej Dammer[b], Miroslav Šoóš[a*]

[a] *Department of Chemical Engineering, University of Chemistry and Technology,*

Technická 3, 166 28 Prague 6 – Dejvice, Czech Republic

[b] *Zentiva, k.s., U Kabelovny 130, 102 00, Prague 10, Czech Republic*

*To whom correspondence should be addressed: Email: dan.trunov@vscht.cz, miroslav.soos@vscht.cz

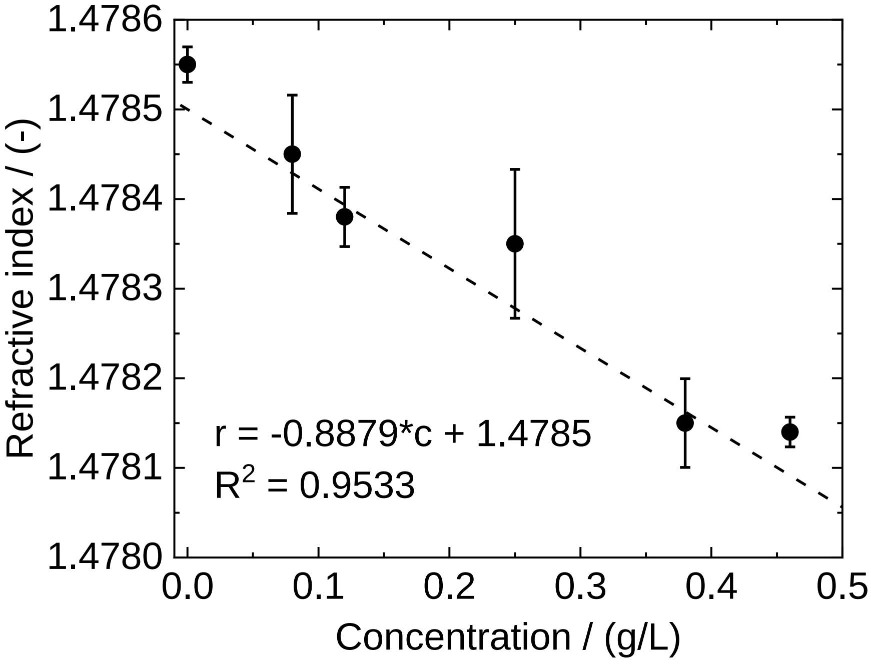

Figure S1 – Refractive index of the dispersed amount IBR in solvent DMSO sokvent as a function of IBR concentration. This lower values of the refractive indexes were in correlation with compounds of lower molecular weight, for example, tetraethoxysilane[1].

Modulated differential scanning calorimetry (mDSC)

mDSC measurements of the materials were collected using DSC 3+ Thermal Analysis System (Mettler Toledo, USA). The samples (5 – 8 mg) were weighted using a covered aluminum pan (40 µL) and measured in air gas flow. The investigation was carried out in the temperature range of 0 to 150 °C with a heating rate of 5 °C/min using ± 1.5 °C amplitude and a 40 s period.

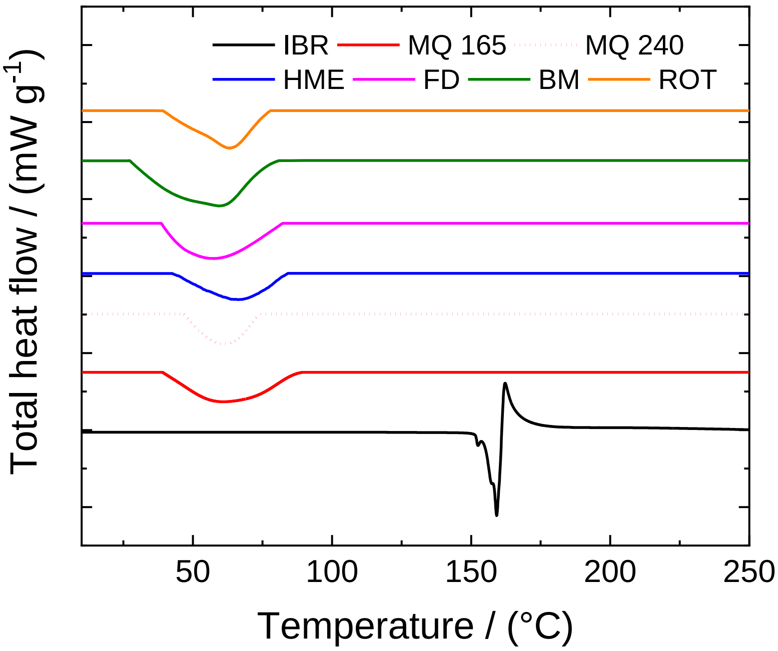

Figure S2 – mDSC thermograms of the drug as well as crystalline and solid dispersions forms of IBR prepared by selected methods of amorphization.

Table S1 – Assignments of 1H NMR spectrum chemical shift of IBR[2]

Atom Position	Type of Atom	^{1}H (ppm)
1	C	-
2	N	-
3	CH	8.24; 8.27
4	N	-
5	C	-
6	C	-
7	N	-
8	N	-
9	C	-
10	C	-
11	CH	7.67

12	CH	7.16
13	C	-
14	CH	7.16
15	CH	7.67
16	O	-
17	C	-
18	CH	7.11
19	CH	7.42
20	CH	7.18
21	CH	7.42
22	CH	7.11
23	CH	4.61; 4.76
24	CHH	2.10; 2.22
25	CHH	1.56; 1.87
26	CHH	3.89; 4.20; 3.08; 2.84
27	N	-
28	CHH	3.08; 3.59; 4.04; 4.52
29	C	-
30	CH	2.45; 5.52
31	O	-
32	CHH	3.54

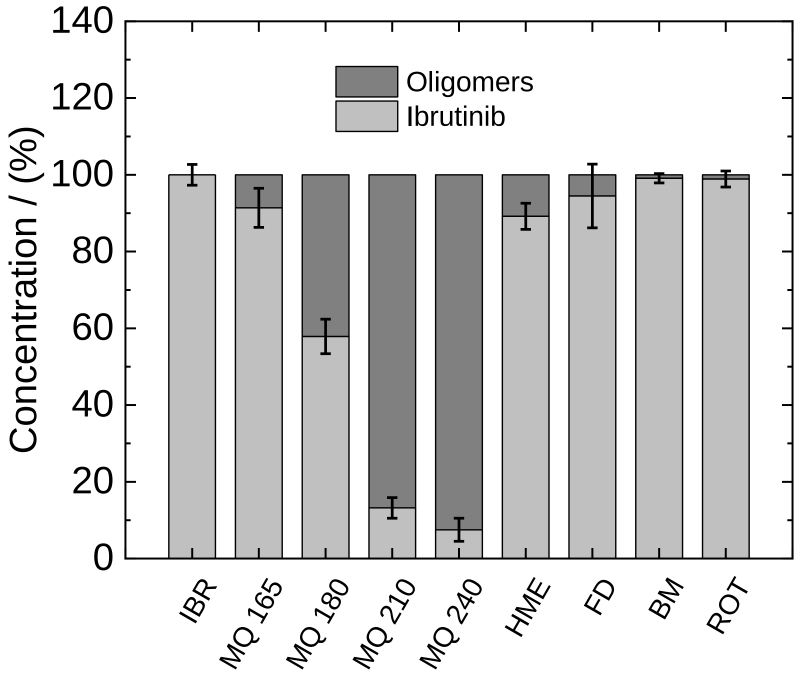

Figure S3 – Concentration of dissolved IBR in the mixture of acetonitrile and water (60:40) obtained using UPLC technique. Amount of oligomers from the UPLC analysis obtained as a mass balance between the initial and measured concentration of IBR.

References

(1) Skvortsov, I. Y.; Varfolomeeva, L.A.; Kulichikhin, V.G. The Effect of Tetraethoxysilane on the Phase State, Rheological Properties, and Coagulation Features of Polyacrylonitrile Solutions. *Colloid J.* **2019**, *81*, 165-175 10.1134/S1061933X19020145.

(2) Vajjha, S.; Bommuluri, V.; Mohan P, K.V.K.; Rumalla, C.S.; Doddipalla, R.; Kaliyaperumal, M.; Korupolu, R.B. Degradation Studies of Ibrutinib Under Stress Conditions: Characterisation and Structural Elucidation of Novel Degradants. *J. Pharm. Biomed. Anal.* **2019**, *172*, 9-17 10.1016/j.jpba.2019.04.010.
